# Voltammetry under a Controlled Temperature Gradient

**DOI:** 10.3390/s100706821

**Published:** 2010-07-14

**Authors:** Jan Krejci, Zuzana Sajdlova, Jan Krejci, Tomas Marvanek

**Affiliations:** BVT Technologies, a.s., Hudcova 533/78c, 612 00 Brno, Czech Republic; E-Mails: tab@bvt.cz (Z.S.); Dzanda@seznam.cz (J.K.Jr.); marvikt@seznam.cz (T.M.)

**Keywords:** Soret phenomenon, electrochemical sensor, thermal diffusion, cyclic voltammetry

## Abstract

Electrochemical measurements are generally done under isothermal conditions. Here we report on the application of a controlled temperature gradient between the working electrode surface and the solution. Using electrochemical sensors prepared on ceramic materials with extremely high specific heat conductivity, the temperature gradient between the electrode and solution was applied here as a second driving force. This application of the Soret phenomenon increases the mass transfer in the Nernst layer and enables more accurate control of the electrode response enhancement by a combination of diffusion and thermal diffusion. We have thus studied the effect of Soret phenomenon by cyclic voltammetry measurements in ferro/ferricyanide. The time dependence of sensor response disappears when applying the Soret phenomenon, and the complicated shape of the cyclic voltammogram is replaced by a simple exponential curve. We have derived the Cotrell-Soret equation describing the steady-state response with an applied temperature difference.

## 1. Introduction

The theory of electrode processes is abundantly described in the literature [1,2]. The electrode response is controlled by electroactive compound transport between the electrode surface and bulk solution. The main mass transport driving forces are electromigration, convection and diffusion. We focus here on convection and diffusion under non-isothermal conditions, where the electrochemical sensor response is controlled by mass transfer between the bulk solution and the layer adhering to electrode surface as well as by the electron transfer kinetics in the electrode reaction. The main process controlling the transfer in the Nernst layer is diffusion. However, diffusion is a relatively slow process. It is much slower than the electrode reaction. The closest neighborhood of an electrode surface is depleted of electroactive compounds. Therefore, the electrode response decreases, as described by the Cotrell Equation [2]. The thickness of the layer where the electrochemical reaction takes place is 0.1 nm to 10 nm. Due to liquid viscosity, it is nearly impossible to use hydrodynamic forces (convection) to accelerate the mass transfer in a layer of this thickness [3].

If very strong hydrodynamic forces are used, *i.e.*, the effective Reynold′s number is extremely high, big fluctuations will cause hydrodynamic noise in the proximity of this layer due to turbulent flow [4,5]. In a layer of a few nanometers adhering to the electrode, pure hydrodynamics are also nearly impossible to use as a tool for the improvement of mass transport between the bulk and the electrode surface. Hence, the question whether another driving force can be applied to increase the mass transport. We reasoned that applying a temperature gradient between the electrode and the solution could serve as a second driving force because thermodiffusion mass transport is independent of the concentration gradient.

The use of temperature to influence the electrode reaction was previously used by Gründler [6], who used a Pt wire heated by Joule heat. Laser pulses have been applied to heat the electrode surface [7]. In another method, the electrode was placed on an insulating layer on metal, which was heated by Joule heat [8,9].

Over the past two decades, attention has been devoted to the improvement of heated electrodes. Grundler and Zerihun have previously tested the functionality of a heated electrode in different arrangements for oxygen measurement, reaching similar cyclic voltammograms as obtained by us [10].

The mass transfer and microfluidics in the proximity of the heated electrode were solved by Frischmuth *et al.*, while the thermodiffusion was not involved in their calculations [11]. Green *et al.* described the basic theory of electrothermally induced fluid flow. The study contains the simulations of the velocity field above the heated electrodes [12]. Oduoza performed electrochemical reaction simulations on the heated platinum wire. He described the mass transfer in terms of convection and hydrodynamics by means of the theory of similarity [13]. The work of Jian-Jun Sun *et al.* describes the graphite heated cylinder electrodes. The mass transport was solved by similarity theory using the similarity numbers, but it does not consider thermodiffusion [14]. Temperature field digital simulations around the wire heated electrodes are presented in another study [15].

Many results increase understanding of the phenomena on heated electrodes, but in many of the studies the methods of non-equilibrium thermodynamics were used. There are also many examples of the analytical use of heated electrodes, which prove the potential of this electrochemical research.

Heated electrodes have been used in many applications including lead detection [16], formaldehyde, methanol and formic acid oxidation [17], anodic stripping voltammetry on heated mercury film electrode [18], electrochemical behavior of cytochrome C [19], identification of DNA damage [20], interaction between DNA and metal complexes [21], electrochemistry of nicotinamide adenine dinucleotide [22], improvement of glucose and maltose sensor specificity [23], electrochemistry of ascorbic acid [24], rutin detection in the nanomolar range [25], thermal stabilization of glucose heated electrodes [26], tool to prevent biochemical fouling on electrodes [27], capillary electrophoresis detectors [28], flow detectors [29], and disposable electrodes [30].

Different concepts were reported regarding heated electrode preparation and use [31–36]. Heated electrodes were combined with electroluminescence in several studies [14,37,38]. A very important application of heated electrodes lies in the emerging field of ionic liquids [39,40]. The comparison of the analytical efficiency of heated electrodes with other methods for detecting lead traces was introduced by Yonghong Li [41].

The heated electrodes seem to be a promising tool in electrochemistry. However, no simple and comprehensive theory of their electrochemical response has been presented yet. All published simulations neglect the fact that a thermal gradient starts thermodiffusion, which is a second mass transport mechanism that can be controlled independently of the concentration by changes in the temperature. This control offers a new parameter for electrochemical measurements, which is not sufficiently appreciated.

The question addresses the technology, which assures the possibility of exact control of temperature gradients at the electrode independently of the concentration. The microelectronic technologies enable the preparation of electrodes on ceramic materials with extremely high specific heat conductivity λ Compare the values provided in Table 1.

The heating resistor can be integrated at a distance of more than ten microns from the electrode. If we realize that the specific heat conductivity of a BeO ceramic is more than two orders higher than that of water, then the temperature of the working electrode surface can be controlled in a very precise manner. The temperature gradient is concentrated in the Nernst layer.

The equation describing the diffusion and temperature are formally the same (parabolic partial equations). Only the parameter α in the temperature equations is about four orders higher than D in the diffusion equations. On the other hand, the transport phenomena connected with non-diagonal elements of the (non-equilibrium thermodynamics) flux matrix are about two orders lower than the diagonal ones.

## Theory

The derivation of the electrode response under isothermal conditions is widely described [1,2]. If a thermal gradient is induced, the system depends on non-equilibrium thermodynamics. The mass transport connected to the thermal gradient was first observed by Ludwig Soret in 1856, which represents a typical example of the coupling of two gradient-cross phenomena. If we suppose that the reaction on the electrode surface is sufficiently fast, then it is possible to assume that the concentration on the electrode surface is zero because the analyte is immediately consumed.

The entropy production σ can be expressed by the following equation (for the general case of several diffusing substances in a continuous, non-isothermal system where no chemical reaction takes place) [43]:

(1) $$\sigma =J_{q}\cdot \mathrm{grad}\frac{1}{T}+\sum_{i=1}^{n}J_{i}\cdot \mathrm{grad}\left(-\frac{\mu_{i}}{T}\right)$$

where:

*σ*: is the local entropy production,
*J_q_*: is the flow of heat,
*T*: is the absolute temperature,
*J_i_*: is the flow of component i in moles per unit area per unit time, and
*μ_i_*: is the chemical potential of component i.

After some simplification, using the Onsager relations of reciprocity [43,3], the final equation for the flow in a binary system can be deduced:

(2) $$J_{s}^{d}=-L_{11}\mu_{\mathrm{ss}}\mathrm{grad}(C_{s})\;-L_{1q}\frac{\mathrm{gradT}}{T}$$

where:

$J_{s}^{d}$: is the flow of the solute relative to the solvent in binary solutions,
*L_ik_*: is a phenomenological coefficient relating to the k^th^ driving force (L_11_, L_1q_ where _q_ is heat),
*μ_ss_*: is the chemical potential of the solute in a binary system, and
*C_s_*: is the molar concentration of the solute in a binary solution.

The total flow of solute depends on two terms. The first term describes the classical or ordinary diffusion proportional to the concentration gradient. The second term describes the thermal diffusion flow induced by the temperature gradient. The coefficient *L_11_μ_ss_* is a classical diffusion coefficient. The coefficient *L_1q_/T* is proportional to the cross non-diagonal coefficient in the classical phenomenological flow matrix in non-equilibrium thermodynamics. The coefficient *L_1q_* is proportional to the solute concentration *C_s_*. Therefore, it is usual to define the thermal diffusion coefficient *D^T^* by the relation:

(3) $$\frac{L_{1q}}{T}=C_{s}D^{T}$$

Equation (2) can then be rewritten in the form:

(4) $$J_{s}^{d}=-D\cdot \mathrm{grad}(C_{s})-C_{s}D^{T}\mathrm{grad}(T)$$

where D is the diffusion coefficient. Thermal diffusion is often characterized by the Soret coefficient s_T_, which is the ratio of thermal diffusion coefficient to the ordinary diffusion coefficient:

(5) $$s_{T}=\frac{D^{T}}{D}=\frac{L_{1q}}{{\mathrm{DC}}_{s}T}$$

Comparing this definition with Equation (4), it is clear that the Soret coefficient describes the equilibrium where the magnitude of flow caused by the gradient of the temperature is exactly equal to the flow caused by the gradient of the concentration [42]. Under these conditions (3), Soret coefficients can be expressed as:

(6) $$s_{T}=-\frac{\mathrm{grad}(\text{ln}C_{s})}{\mathrm{gradT}}$$

It was found that the thermal diffusion coefficient is smaller by a factor from 100 to 1,000 than the ordinary diffusion coefficient for electrolytes, non electrolytes and gases [43]. The concentration gradient is relatively small unless the thermal gradient is very large. However, a completely different situation is solved here. Due to the very high thermal conductivity of ceramics, the thermal gradient in the liquid adjacent to the electrode surface reaches very high values of the temperature gradient, and in addition, the temperature gradient can be adjusted independently of the solute concentration *C_s_*. The thermodiffusion can play an important role in the enhancement of the mass transfer in the proximity of the surface of the electrode. Other new results can be found in the literature [44,45].

## Theory of the potential steps at an planar electrode including thermo diffusion mass transport

Consider the reaction [1] *O* + *ne* ⇔ *R*, which is started by a potential step of any magnitude. An experiment begins at *t* = *0* and at a potential at which no current flows. The potential E is instantaneously changed to a value anywhere on the reduction wave. Very rapid charge transfer kinetics are assumed here, so *O* and *R* are always in equilibrium at the electrode surface, with the concentration ratio given by the Nernst equation:

(7) $$\theta =\frac{C_{O}(0,t)}{C_{R}(0,t)}=\text{exp}\frac{\mathrm{nF}({E-E}^{\circ })}{{\mathrm{RT}}_{1}}$$

where:

*C_O_(x,t)*: is the concentration of the oxidized compound (reducible),
*C_R_(x,t)*: is the concentration of the reduced compound (oxidable),
*n*: is the number of electrons in the electrode reaction,
*F*: is the Faraday constant,
*E*: is the electrode potential,
*E^0^*: is the standard electrode reaction potential,
*R*: is the gas constant,
*T_1_*: is the temperature of the electrode surface, and
*t*: is the time.

**Figure 1.**
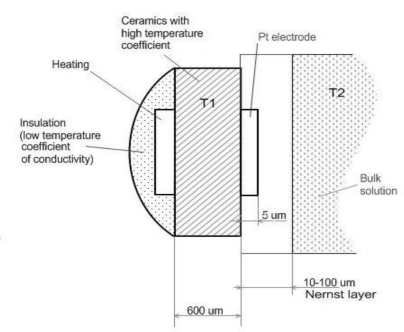
Schematics of temperature gradient application on planar electrode realized on ceramics and immersed in the solution analyzed.

Not only a voltage but also a temperature gradient is applied there. The planar electrode (Figure 1) is maintained at temperature *T_1_*, and the solution surrounding the electrode is maintained at temperature *T_2_* by an external thermostat.

The problem can be specified as follows:

- The electrode is planar and has a surface area A. Only diffusion along the x-axis perpendicular to the electrode surface needs to be considered. The electrode is sufficiently large so that the edge effects can be neglected.
- The solution is initially homogenous. Specifically, the initial concentration of *O* and R are *C_O_*(*x,0*) = *C_O_* and *C_R_*(*x,0*) = *0* for all values of x at time *t* = *0*.
- The electrolysis cell is sufficiently large that the bulk concentrations of O and R are unchanged from the initial values even after electrolysis has been running for a certain time. In other words, *C_O_*(*x,t*)→*C_O_*, *C_R_*(*x,t*)→*0* while *x*→∞.
- For every O molecule consumed, an R molecule is formed; in other words, the fluxes *J_O_*(*0,t*) and *J_R_*(*0,t*) of O and R at the electrode surface are equal and opposite in sign: *J_O_*(*0,t*) = −*J_R_*(*0,t*).
- The electron-transfer reaction is very fast, such that *O* and R are always in equilibrium at the electrode surface with the concentration ratio given by the Nernst Equation (7).
- The system can be treated as two binary systems of (solvent and *O*) and (solvent and *R*). There is no interaction between *O* and *R*.
- The reaction takes place at the electrode surface, the temperature of which is *T_1_*.

There is only one difference from the classical solution. The flux depends on the temperature gradient and on the concentration gradient:

(8) $$J(x,t)=-D\frac{\partial C(x\text{,}t)}{\partial x}-D.C(x,t).s_{T}.\frac{\partial T(x,t)}{\partial x}$$

We suppose that the system is in thermal equilibrium, which means:

(9) $$\frac{\partial T(x,t)}{\partial x}=\alpha .\Delta T=\alpha (T_{2}-T_{1})$$

where α is a proportionality constant. The electrode is heated (*T_1_* > *T_2_*). The final diffusion equations are derived using Fick’s laws:

(10) $$\frac{\partial }{\partial t}C_{\mathrm{Ox}}\;(t,x)=D_{\mathrm{Ox}}\left(\frac{\partial^{2}}{{\partial x}^{2}}C_{\mathrm{Ox}}(t,x)\right)+\sigma_{\mathrm{Ox}}\left(\frac{\partial }{\partial x}C_{\mathrm{Ox}}(t,x)\right)$$

(11) $$\frac{\partial }{\partial t}C_{\text{Re}d}(t,x)=D_{\text{Re}d}\left(\frac{\partial^{2}}{{\partial x}^{2}}C_{\text{Re}d}(t,x)\right)+\sigma_{\text{Re}d}\left(\frac{\partial }{\partial x}C_{\text{Re}d}(t,x)\right)$$

where *σ_Ox_* = *D_Ox._s_T,Ox._α*(*T_1_−T_2_*) and *σ_Red_* = *D_Red._s_T,Red._α*(*T_1_−T_2_*). *D_Ox_* and *D_Red_* are diffusion coefficients for *O* and *R*, respectively. *s_T, Ox_* and *s_T,Red_* are the Soret coefficients for *O* and *R*, respectively.

The definition of the problem implies the boundary conditions at infinity:

(12) $$\begin{array}{l} \underset{x\to \infty }{\text{lim}}C_{\mathrm{Ox}}\;(t,x)=C_{0},\\ \underset{x\to \infty }{\text{lim}}\;C_{\text{Re}d}(t,x)\;=\;0. \end{array}$$

The boundary conditions at the surface of electrode are expressed through the flux balance, which means that the flux of the oxidized compound is in equilibrium with the flux of the reduced compound. The second condition states that all oxidized compounds are immediately changed to the reduced form at the electrode surface:

(13) $$D_{\mathrm{Ox}}({\left(\frac{\partial }{\partial x}C_{\mathrm{Ox}}(t,x)\right)}_{x=0})+\sigma_{\mathrm{Ox}}C_{\mathrm{Ox}}(t,x)=-D_{\text{Re}d}({\left(\frac{\partial }{\partial x}C_{\text{Re}d}(t,x)\right)}_{x=0})+\sigma_{\text{Re}d}C_{\text{Re}d}(t,x)$$

(14) $$\frac{C_{\mathrm{Ox}}(t,0)}{C_{\text{Re}d}(t,0)}=e^{\left(\frac{\mathrm{nF}(E-E^{0})}{{\mathrm{RT}}_{1}}\right)}$$

We can denote the exponential term as θ:

(15) $$\theta (T_{1})=e^{\left(\frac{nF(E-E^{0})}{RT_{1}}\right)}$$

The initial conditions are: *C_Ox_*(*0*,*x*) = *C_0_*, *C_Red_*(*0*,*x*) = *0*.

In the most simple case it is possible to consider *D_Ox_* = *D_Red_* = *D* and *σ_Ox_* = *σ_Red_* = *σ*.

The application of the Laplace transform to equations (10) and (11) in consideration of conditions (12) yields:

(16) $$i=-i_{0}(\frac{e^{-\mathrm{Bt}}}{\sqrt{\pi t}}+\sqrt{B}.\mathrm{erf}(\sqrt{\mathrm{Bt}}))$$

where:

(17) $$i_{0}={\mathrm{nFAC}}_{0}\frac{\sqrt{D}}{1+\theta (T_{1})},$$

(18) $$B=D.{\left[s_{T}.\alpha (T_{1}-T_{2}\right]}^{2},$$

and *i_0_* is steady state current.

Equation (16) has very interesting impacts. At zero temperature gradient at the electrode, *T_1_* = *T_2_*, the coefficient *B* = *0*, *erf* (*0*) = *0*, and it becomes the classical Cotrell equation.

If *T_1_* ≠ *T_2_* then *B* ≠ *0* and $\frac{e^{-\mathrm{Bt}}}{\sqrt{\pi t}}$ decreases quickly, while the second term exponentially tends to $\sqrt{B}$, which is time independent. If the temperature gradient is present, the current approaches a steady state value given by equation:

(19) $$i=n.F.A.C_{0}\frac{D}{1+\theta (T_{1})}.s_{T}.\alpha .(T_{1}-T_{2})$$

where *θ*(*T_1_*) stresses the fact that due to high temperature conductivity of ceramic substrate of the sensor the *T_1_* temperature of the electrode surface is known.

## 2. Experimental Section

**Figure 2.**
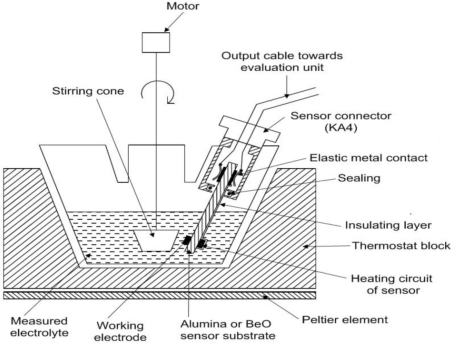
Schema of the Soret system (the gap between the cone and electrode surface is 1 mm).

Measurements were performed in a device consisting of a glass cell TC1, conic stirrer and connector KSA1 and electrochemical sensor AC1.W2.RS (H,T) (BVT Technologies, Czech Republic). The AC1.W2.RS (H) electrochemical sensor bears platinum working and auxiliary electrodes, a pseudo-reference silver electrode and a heating circuit. The cell TC1 was placed in a small thermostat TK-1 (KEVA, Czech Republic). The whole system schematic is shown in Figure 2.

## 3. Results and Discussion

Formula (16) plays a key role in this new method. If the temperature difference is zero, then the parameter B is also zero. Then, the exponential term vanishes, and the second term vanishes as well. The formula changes to the normal Cottrell equation. However, if the temperature difference is nonzero, then B is also nonzero. The influence on Equation (16) is dramatic.

**Figure 3.**
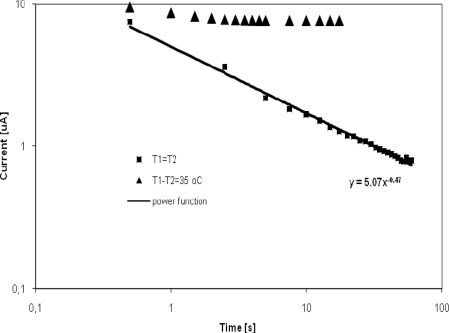
The time dependence of sensor output current; comparison of Cottrell response (▪ *T_1_* = *T_2_*) and response with thermo diffusion (▴*T_1_* − *T_2_* = 35 °C)

The classical Cottrell term decreases with an exponential rate. The second term, containing the error function, converges very fast to the value of the square root of B. This value is constant and time independent. The situation is shown in Figure 3.

The experimental exponent −0.47 is close to the theoretical value −0.5 in the case of no temperature gradient; however, if the temperature is implied the response stabilizes to a steady state current *i_0_.s_T_.α*. (*T_1_−T_2_*). The current is stabilized in 2 s in comparison with pure diffusion where the current is not stabilized after 60 s. As the Cottrell term vanishes with an exponential rate, after a short time the response of the sensor is controlled by the thermo diffusion term only.

**Figure 4.**
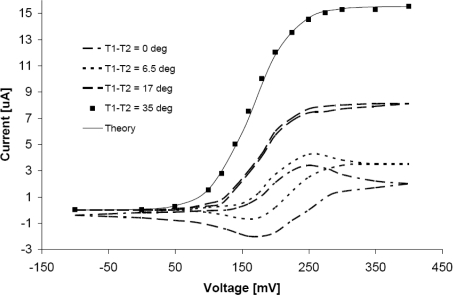
The change of cyclic voltammetry with application of thermal gradient. The ▪ points are experimental points, and the continuous line is the theoretical run of function 1/(1 + *exp*((*nF.*(*E−E^0^*)/*RT*)).

In the case of cyclic voltammetry measurements the response is changed dramatically. The higher the temperature difference between electrode and solution is, the narrower oxidation and reduction wave is until it completely disappears. This situation is visible in Figure 4.

At a temperature difference of 35 °C, the reduction and oxidation curves are the same. The thermodiffusion under such conditions is sufficiently strong that the first term in (16) can be omitted. The cyclic voltammetry can be described by (19), where the voltage is in the term *θ* (*T_1_*) = *exp* (*nF*(*E−E^0^*)/*RT_1_*).

The complicated shape of the cycling voltammetry disappears and at a temperature difference of 35 °C only a very simple curve remains, which can be analyzed in a simple manner using formula (19). The agreement between experiment and theory is again very good. The same or very similar experimental results were also obtained in [21,22,37,35].

It is also possible to see that the curves shift to the left. The slope of curve is also increased. This phenomenon can be understood if we realize that the reaction takes place on the electrode surface, the temperature of which is growing. The solvent is maintained at the temperature *T_2_*, and the temperature of the electrode T_1_ is increased.

**Figure 5.**
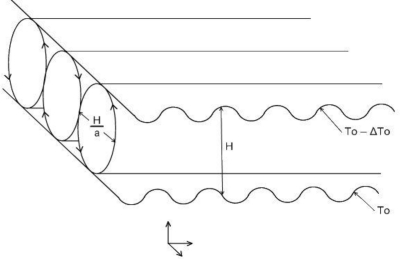
Convective flow above the heated surface.

Liquid flow in the vicinity of or above the heated surface can be solved using a different point of view. Lorenz [46] studied a hydrodynamic system that was an approximation of flow above the earth surface. He found out that deterministic differential equations may provide a solution that seems to be random noise with nondeterministic behavior. His work led to the “strange attractor” concept [47]. A set of convective flows originates in the layer above the heated surface (see Figure 5) [48].

Regarding the electrochemical measurements, the thermally induced microconvection is focused in a Nernst layer of a few microns in thickness. Therefore, the temperature gradient is applicable for micro to nano mixing, which is localized in the very vicinity of the working electrode. The experiments performed did not answer whether the prevailing mechanism is microconvection or pure thermodiffusion.

**Figure 6.**
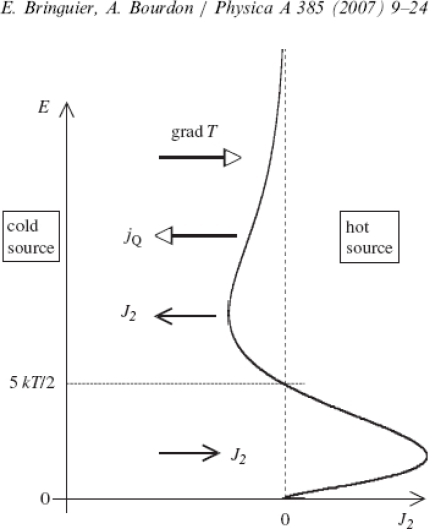
Particle and heat flows in a monoatomic gas subjected to a temperature gradient at steady state. The spectral particle-current density *J_2_*(*E*) shows that “cold” molecules (*E* < 5 kT/2) go to the hot region (*J_2_*(*E*) > 0), and vice versa; the net particle-current density *∫J_2_*(*E*)*dE* vanishes. The heat-current density j_Q_ is ∫*EJ_2_* (*E*) *dE*. Reprinted from *Physica A,* **385,** *E.* Bringuier, *A.* Bourdon, Kinetic theory of colloid thermodiffusion, 9–24, Copyright (2007), with permission from Elsevier.

Bringuier [44] published the kinetic theory of colloid thermal diffusion. Figure 6 presents his very instructive illustration of flow behavior under a temperature gradient. This figure shows that the application of a temperature gradient significantly changes the flow at the surface of an electrode. Even though these results were developed for monatomic gases it can give a picture of nature of phenomena that can occur at electrode surface as well.

## 4. Conclusions

The time dependence of the sensor response disappears when applying the Soret phenomenon. The sensor current stabilizes to constant value, and the complicated shape of cyclic voltammogram is replaced by simple exponential curve. The Cotrell-Soret equation describing the steady-state response with applied temperature difference is then:

(20) $$i=-n.F.A.C_{0}.D.s_{T}.\alpha ,(T_{1}-T_{2})\frac{1}{1+e^{\frac{\mathrm{nF}(E-E^{0})}{{\mathrm{RT}}_{1}}}}$$

where *T_1_* is the temperature of the sensor, and *T_2_* is the temperature of the liquid.

If a voltammetric scan is performed in a broad potential window, one can identify a current value where thermodiffusion is in equilibrium with the electrode reaction: *i_0_* = *−n.F.A.C_0_.s_T_.α.*(*T_1_−T_2_*).

Then Equation (20) allows computation of *E^0^* at temperature *T_1_*:

(21) $$\frac{i}{i_{0}}=\frac{1}{1+e^{\frac{\mathrm{nF}(E-E^{0})}{{\mathrm{RT}}_{1}}}}$$

## Figures and Tables

**Table 1.** Values for thermal conductivity (**λ)**, thermal diffusion (**α)** and diffusion coefficient for small molecules like water.

**Material**	**λ**	**α**	**D**
	**W/m·K**	**m^2^/s**	**m^2^/s**
Water at 25 °C	0.6	0.14 × 10^−6^	10^−10^–10^−12*^
Al_2_O_3_ ceramic	35	8.8 × 10^−6^	
BeO	180	89 × 10^−6^	
Ag	420	172 × 10^−6^	

* Low molecular weight compounds.

## List of Symbols

A: area of electrode surface
C_O_(x,t): the concentration of oxidized compound (reducible)
C_R_(x,t): the concentration of reduced compound (oxidable)
C_s_: molar concentration of solute in a binary solution
C_0_: initial molar concentration
D_Ox_, D_Red_: diffusion coefficients for Ox and Red, respectively
E: cell potential
E^0^: standard redox potential
F: Faraday constant
i: electric current
i_0_: steady state current
J: molar flux density
J_i_: flow of component i in moles per unit area per unit time
J_q_: is a flow of heat
J_s_^d^: flow of solute relative to solvent in binary solutions
L_ik_: phenomenological coefficient relating to the k^th^ driving force (L_11_, L_1q_ where _q_ is heat)
λ: specific heat conductivity
n: number of electrons in electrode reaction
R: gas constant
s_T, Ox_ and s_T,Red_: Soret coefficients for Ox and Red, respectively
t: time
T: absolute temperature
T_1_: temperature of electrode surface
T_2_: temperature of liquid
α: proportionality constant
μ_i_: chemical potential of component i
μ_ss_: chemical potential of solute in a binary system
